# Direct Resazurin Microplate Assay in Drug Susceptibility Testing of Smear-Positive Sputum Samples against *Mycobacterium tuberculosis*

**DOI:** 10.21315/mjms2018.25.6.6

**Published:** 2018-12-28

**Authors:** Noor Izani Noor Jamil, Wan Nor Amilah Wan Abdul Wahab, Ibyhaslin Alyani Ali, Mohammad Lukman Yahaya

**Affiliations:** School of Health Sciences, Health Campus, Universiti Sains Malaysia, 16150 Kubang Kerian, Kelantan, Malaysia

**Keywords:** Mycobacterium tuberculosis, smear-positive sputum, resazurin microplate assay, drug susceptibility testing, isoniazid, rifampicin

## Abstract

**Background:**

A new direct microplate-based colorimetric drug susceptibility test that omits the initial isolation of *Mycobacterium tuberculosis* from sputum specimens was evaluated.

**Methods:**

A total of 51 *M. tuberculosis* acid fast bacilli (AFB) smear-positive sputum specimens were inoculated directly into drug-free and serial dilutions of drug-containing Middlebrook 7H9 broth media. With this direct resazurin micro plate assay (REMA) method, resazurin dye was used as a growth indicator in microplate wells. The minimum inhibitory concentrations (MIC) of isoniazid (INH) and rifampicin (RIF) were compared with those of the ‘gold standard’ absolute concentration method (ACM). The turnaround time (TAT) of the direct REMA and the ACM were also determined.

**Results:**

At the selected cut-off points (INH: 0.0625 μg/mL; RIF: 0.125 μg/mL), good drug susceptibility test results were obtained for INH and RIF with an average sensitivity, specificity and accuracy of 90%, 100% and 97%, respectively, with a TAT of 15 days. The REMA method also correctly classified the resistant isolates with positive predictive values of 95% and negative predictive values of 98% for the two drugs.

**Conclusions:**

The direct REMA was reliable in routine diagnostic laboratories for the drug susceptibility testing of *M. tuberculosis* and the rapid detection of multi-drug-resistant tuberculosis.

## Introduction

Tuberculosis (TB) continues to be a major public health problem in developed and developing countries. An estimated two billion people have the disease (1). The highest prevalence of TB cases has been found in Asia and Africa, which accounted for 85% of all cases in 2013 and approximately 78% of all related deaths (2). There were an estimated 1.4 million TB deaths in 2015. Although the number of TB deaths fell by 22% between 2000 and 2015, the disease remained one of the top 10 causes of death worldwide in 2015 (3–4).

Over the past five years in Malaysia, there has been a steady increase in the number of TB notifications: approximately 52–81 per 100,000 people between 2013 and 2017, with an increased rate of 1%–7% annually until 2016 (5–6). In 2015 and 2016, 24,071 and 25,739 cases, respectively, of all forms of TB were notified in Malaysia (3–6). The development of multi-drug resistant *Mycobacterium tuberculosis* (MDR-TB) and the recent emergence of extensively drug resistant *M. tuberculosis* (XDR-TB) are major obstacles to the treatment and management of TB. The World Health Organization reports on the increasing prevalence of anti-TB drug resistance in the world have confirmed that drug-resistant *M. tuberculosis* is ubiquitous and that the incidence of MDR-TB and XDR-TB has also increased alarmingly (2, 4).

The MDR-TB prevalence in Malaysia was 0.3%–1.3% from 2004 to 2011. It dropped to 0.9% at the end of 2013 because of improved preventive and control measures (2). According to the 2014 statistics, of the notified TB cases in Malaysia, 80, i.e. 0.4%, were MDR-TB (2). In 2015, the estimated MDR-TB cases in Malaysia were 98, i.e. 0.53% of all notified cases (3). However, no recent systematic and detailed data are available regarding the prevalence of drug-resistant *M. tuberculosis* in Peninsular Malaysia. One of the reasons for the paucity of information on antibiotic resistance patterns in *M. tuberculosis* is the lack of a quick, simple and affordable technique that can be used in all laboratories to determine antibiotic susceptibility. The available methods, namely the absolute concentration method (ACM), the proportion method, the BACTEC and the Etest, are tedious, lengthy and/or expensive for antibiotic susceptibility determination purposes.

Inexpensive rapid colorimetric methods for the determination of susceptibility to first-line drugs and MDR-MTB are currently available. Multiple samples can be tested simultaneously; however, the assays require the use of pure cultures from clinical samples. These methods have long turnaround times (TATs) for obtaining the final sensitivity result because of the need for the initial isolation of the organisms from the samples prior to the colorimetric assay.

Therefore, the determination of antibiotic susceptibility directly from clinical samples, without the initial isolation of the *Mycobacterium*, would overcome this limitation and significantly shorten the TAT for isolation and drug sensitivity. A modified colorimetric resazurin microplate assay (REMA) was performed to determine the susceptibility of *M. tuberculosis* to first-line drugs directly on acid fast bacilli (AFB) smear-positive sputum samples without the prior isolation of the organisms from the samples.

## Materials and Methods

### Sample Collection and Processing

A total of 51 AFB-positive sputum samples were collected from the Hospital Universiti Sains Malaysia and Hospital Raja Perempuan Zainab II in Kelantan, Malaysia over a period of 18 months. This study was granted ethical approval by the Human Research Ethics Committee of Universiti Sains Malaysia [Reference No: USMKK/PPK/JK EP (M)-USM 191.3 (4)]. AFB-positive sputum specimens were processed for decontamination by the standard N-acetyl-L-cysteine sodium hydroxide (NALC-NaOH) method within 24 h. Decontaminated sputum samples were aliquoted and processed for inoculation onto an Ogawa medium (Oxoid, UK) in duplicate for the primary isolation of the mycobacteria and drug sensitivity testing by the REMA method. The tube media were observed weekly up to 8 weeks for the presence of growth. Colonies from the tube media were confirmed for the presence of AFB by Ziehl-Neelsen (Z-N) staining.

### Inoculum Preparation

An inoculum of *M. tuberculosis* H37Rv was prepared from the log phase culture, which had been incubated for approximately 21–28 days. A loop full of colonies was taken and transferred into sterile Bijou bottles containing 2.5 mL Middlebrook 7H9-S broth. The suspension was vortexed to break the clumps, and 2 mL supernatant was then transferred to sterile flat-bottomed glass test tubes and left to sediment for 15 min. The turbidity of the suspension was measured and adjusted to McFarland 1.0 where the approximate per mL bacterial suspension was 3.0 × 10^8^. The suspension was then diluted with Middlebrook 7H9-S broth to provide the desired standard inoculum: 2.0 × 10^3^ CFU/mL.

### Mycobacterium tuberculosis Identification

The growing AFB, which resembled *M. tuberculosis* and the non-tuberculosis *Mycobacterium* (NTB), were subjected to further identification at the National Public Health Laboratory, Sungai Buluh, Selangor, Malaysia. The identification of *M. tuberculosis* from all of the positive sputum specimens was performed by a DNA probe test (Gen-Probe, Inc., San Diego, CA) according to the manufacturer’s instructions.

### Drug Susceptibility Testing by a Direct Resazurin Micro Plate Assay

The colorimetric microplate method was used to test the samples directly for drug sensitivity. Briefly, decontaminated samples were directly cultured in 24-well tissue-culture plates in Middlebrook 7H9 broth containing OADC and PANTA (Becton-Dickinson, USA). For each sample, 12 wells were used. In the control wells, no drug was added. Each of the remaining wells contained one of two drugs: isoniazid (INH) (Sigma-Aldrich, USA) or rifampicin (RIF) (Sigma-Aldrich, USA). The concentrations were 0.25–0.0078 μg/mL and 0.5–0.0156 μg/mL for the INH and RIF, respectively. The direct REMA was performed in duplicate. The wells containing broth media and bacterial suspension MTB H37Rv served as the positive controls, and the wells containing only broth media were the negative controls. Parafilm-sealed plates were then incubated at 37 °C. After 10 days of incubation, 50 uL resazurin (Acros Organics NV, Belgium) at a dilution of 1 mg/mL was added to the control wells. The growth of *M. tuberculosis* could then be observed as a change in the colour of the dye because of the oxidation-reduction mechanism.

The colour changes in the positive control wells were observed to determine the subsequent addition of resazurin dye in the test wells. The MIC was defined as the lowest drug concentration that prevented a colour change of blue to pink in the resazurin in the test wells. The amount of time for the return of results was also noted for the direct REMA method. The TAT was defined as the time between the date on which the samples were processed and the date on which the results were available.

### Drug Susceptibility Testing by the Absolute Concentration Method

The ACM has been the ‘gold standard’ for determining anti-TB drug susceptibility. *M. tuberculosis* H37Rv (ATTC 27294) was used as a control strain. The ACM was performed on a Lowenstein–Jensen (LJ) medium in tubes according to the National Public Health Library Standard Procedure. In brief, the LJ tube media were incorporated with graded concentrations of INH and RIF. The recommended critical concentrations were 0.1, 1.0 and 5.0 μg/mL; for the INH and 50 μg/mL for the RIF. The drug-incorporated tube media were inspissated at 80 °C for 50 min. The media were inoculated with the standardised inoculum (2 × 10^3^ CFU/mL) from pure culture isolates. The culture media were incubated at 37 °C in the presence of 5%–10% CO_2_ until growth was observed. *M. tuberculosis* H37Rv was used as the standard reference, i.e. the control. Strains exhibiting growth greater than 1%, or more than 20 colonies, were classified as resistant. The minimum inhibitory concentrations (MIC) was defined as the lowest drug concentration that reduced the growth of the test organism to 1% or less, fewer than 20 colonies, when compared to the control slope. The concentration in which a majority of strains were sensitive was taken as the MIC. The TAT of the test was also determined.

### Data Analysis

The sensitivity, specificity, accuracy and predictive values for the susceptibility (PVsS) and the predictive values for the resistance (PVsR) of the direct REMA of the clinical samples in comparison to those for the ACM were calculated with the formula below (7):

$$\begin{array}{l} \text{Sensitivity}=\frac{\text{true resistant}}{\text{true resistant}+\text{false sensitive}}\times 100\%\\ \text{Specificity}=\frac{\text{true sensitive}}{\text{true sensitive }+\text{false resistant}}\times 100\%\\ \text{Accuracy}=\frac{\text{true resistant}+\text{true sensitive}}{\text{true resistant}+\text{false resistant}+\text{true sensitive}+\text{false sensitive}}\times 100\%\\ \text{PVS}=\frac{\text{true sensitive}}{\text{true sensitive}+\text{false resistant}}\times 100\%\\ \text{PVR}=\frac{\text{true resistant}}{\text{true resistant}+\text{false}\;\text{sensitive}}\times 100\% \end{array}$$

True resistance was defined as the MIC values equal to or exceeding the cut-off point concentration by the ACM. False resistance was defined as the MIC values that were resistant by the REMA but susceptible by the ACM. True sensitivity was defined as the MIC values that were less than the cut-off point concentration by the ACM, and false sensitivity was defined as the MIC values that were susceptible by the REMA but resistant by the ACM.

## Results

### Minimum Inhibitory Concentrations of Isoniazid and Rifampicin by the Direct Resazurin Microplate Assay and Absolute Concentration Methods

The 51 sputum samples that tested positive for *M. tuberculosis* were analysed. The MICs of the INH and the RIF on the *M. tuberculosis* isolates by the direct REMA method are shown in Table 1. A visual reading of the direct REMA, which was the best method for distinguishing resistant and susceptible strains, indicated that the MIC cut-off points for the INH and the RIF were 0.0625 μg/mL and 0.125 μg/mL, respectively. The value was selected on the basis of the agreement in the number of sensitive strains by the REMA compared to that by the ACM, with which most strains were found to be sensitive (Table 1).

### Susceptibility of Mycobacterium tuberculosis to Isoniazid and Rifampicin by the Direct Resazurin Microplate Assay Method

Table 2 presents a comparison of the number of resistant and susceptible *M. tuberculosis* strains observed by the ACM and the direct REMA on the basis of the defined cut-off point concentration. Of the 51 *M. tuberculosis* isolates tested with the ACM, 41 were susceptible to INH, and 10 were resistant. In addition, 43 isolates were susceptible to RIF, and eight were resistant. In the direct REMA, 40 isolates were susceptible to INH, and nine were resistant. There were two discordant results. One isolate was found to be resistant by the ACM but susceptible by the direct REMA, and another isolate was susceptible by the ACM but resistant by the direct REMA at the predetermined cut-off point. Forty-three isolates were susceptible to RIF and seven isolates were resistant, with only one discordant result (resistant by ACM but susceptible to RIF) at the predetermined cut-off point (Table 2). The direct REMA could correctly identify almost all of the true INH and RIF resistant isolates; thus, the overall performance of the direct REMA for the two drugs was acceptable. The susceptibility results by the direct REMA for the 51 AFB smear-positive *M. tuberculosis* sputum samples and the MTB H37Rv strain (ATCC 27294) were available within a mean TAT of 15 days of incubation. The susceptibility results from the ACM were available only after 3–6 weeks.

### Sensitivity, Specificity, Accuracy and Predictive Values by the Direct Resazurin Microplate Assay Method

The performance characteristics of the REMA method for the detection of resistance to RIF and INH, the two drugs used in the study, are shown in Table 3. The direct REMA method exhibited very good performance characteristics for detecting resistance to the two drugs, with 90% sensitivity, 98% specificity, 96% accuracy, 90% PVR and 98% PVS for the INH because of the two discordant results. For the RIF, 88% sensitivity, 100% specificity, 98% accuracy, 100% PVR and 98% PVS were obtained because there was only one discordant result. The high PVsR for the INH (90%) and the RIF (100%) were observed by the direct REMA method. Similarly, very good PVsS (98%) for both the INH and the RIF were also obtained by the direct REMA method. These results indicate that the direct REMA method was very sensitive for detecting true resistant and true susceptible strains for both drugs.

### Multi-drug-resistant Mycobacterium tuberculosis Prevalence by the Direct Resazurin Microplate Assay Method

The prevalence of MDR-TB by the ACM and the direct REMA method was also determined. The direct REMA method correctly classified five of six mono-resistant TB isolates. However, the direct REMA method, like the standard ACM, could classify all six MDR-TB isolates correctly. The prevalence of mono-drug-resistant TB (10%) and MDR-TB (12%) by REMA was in close agreement with the results of the ACM (Table 4). Thus, the direct REMA was reliable for determining the resistant isolates for the INH and the RIF.

## Discussion

The purpose of this study was to compare the reliability of the direct REMA method to the ACM for the susceptibility testing of *M. tuberculosis* to INH and RIF without the prior isolation of *M. tuberculosis* in sputum specimens.

In Malaysia, the drug susceptibility testing (DST) is performed in the Malaysian National Public Health Laboratory, Selangor. It is based on the conventional culture method, the ACM, which is lengthy and laborious. It requires at least four weeks of incubation before an isolate can be reported as resistant or susceptible. The longer time needed for obtaining results by this standard method is hindering the control and timely treatment of TB. Thus, the development of rapid and reliable diagnostic assays is a priority for the management of the disease. The recent rapid phenotypic colorimetric methods that use liquid media and an indicator to produce a colour change by a redox reaction have been described as successful for DSTs (8). Preliminary studies using colorimetric assay methods have shown good correlation with the conventional standard methods (9–10), and the REMA plate method has proved reliable for the detection of MDR-TB with first- and second-line TB drugs (11–12). However, these colorimetric assays used pure cultures from clinical samples. The TAT for the final sensitivity result was therefore longer because the performance of the test required the initial isolation of the organisms from the samples. However, the current study, using the direct REMA method, yielded the optimal overall efficiency, including a reduced TAT, for the first-line anti-TB drugs INH and RIF. As with most other direct DST methods, tests can be performed and colour changes interpreted relatively easily with the direct REMA method.

In the current study, the direct REMA method exhibited similar diagnostic ability to the ACM by enabling the clear and easier separation of arbitrarily chosen MIC values. The defined cut-off values for the two drugs, INH and RIF, allowed for the differentiation of the resistant and the susceptible strains. The results obtained from the direct REMA were as encouraging as those of previous studies, given that no major discordance was found regarding the detection of resistance to the two drugs. The cut-off values were observed to be relatively lower than those of previous DST studies (10–11, 13). The differences might have been the result of the technical variations in the tests and the different susceptibility patterns and distributions of the resistant isolates from each study (13). However, the MIC was similar to that of a local study even though non-clinical samples were used (14). The lower cut-off value was in concordance. It was supported by the assertion in previous studies that the current cut-off value for first-line anti-TB drugs is overly optimistic (13, 15). The proposed cut-off values were similar to those in this study. It is worth noting that the cut-off values chosen for this study are very effective for distinguishing between susceptible and resistant isolates.

The sensitivity and specificity to INH (90% and 98%, respectively) and RIF (88% and 100%, respectively) were as good as those observed with other direct methods (16). The minor discrepancies observed with the ACM might be the result of differences in the substantial distribution of drug-resistant isolates in the studied population, the inoculum size, and the incubation period variations that caused drug degradation (17). The accuracy of the direct REMA for the INH (96%) and the RIF (98%) were good. The results were similar to those of previous direct colorimetric studies (13). The minor observed discordance can be overcome by adjusting the cut-off values and increasing the sample sizes for the resistant strains and the studied population. The average TAT for obtaining results was 15 days. This was shorter than that for the conventional ACM, which requires a minimum of 3–6 weeks before the first results can be reported (16). Despite the similarity in the sensitivity and specificity of the direct methods, the average TAT for the susceptibility result by direct REMA was relatively shorter than the reported mean TAT of 15–28 days for most other direct methods, specifically the nitrate reductase assay and the microscopic observation drug susceptibility methods (16, 18).

The PVsR and PVsS were also evaluated because both types of values are useful for determining sensitivity and specificity results. In addition, they are dependent on the tested population and disease prevalence. In the current study, the classification of the prevalence of a resistant MTB strain by the direct REMA method was high for mono-resistant TB (10%) and MDR-TB (12%). The direct REMA method thus performed well in detecting mono-drug resistant TB and MDR-TB to INH and RIF, given that similar results were observed with the ACM method. The national estimate for MDR-TB is approximately 1%. It is possible that the high MDR-TB observed in this study might have been the result of the small number of isolates that were tested. Until and unless systematic and detail data on the prevalence of drug resistant *M. tuberculosis* in peninsular Malaysia become available, the prevalence pattern might remain undetermined. Nevertheless, studies done in countries with high resistance prevalence rates have yielded high PVsR (19–20). The direct REMA performed similarly to the ACM. This suggests that it might be a reliable and effective method for clinical laboratories to use for the rapid detection of MDR-TB strains. The efficiency of the assay method can be further improved to produce reliable results, thereby providing efficient tools for the rapid and accurate testing of susceptibility to firstline drugs. This would be valuable for the laboratories of under-resourced countries where MDR-TB is widespread and often fatal.

Conventional drug susceptibility methods are still the mainstay in the detection of drug resistant isolates in most countries. In summary, the results of the direct REMA method were comparable to those of the ACM. The direct REMA method showed good sensitivity and specificity in the DST for *M. tuberculosis*. The direct REMA therefore offers an alternative method for the determination of DSTs. It is relatively cheaper, simpler and faster. It overcomes the limitations of culturing and isolating the mycobacteria, thus requiring a much shorter TAT for drug susceptibility testing.

## Figures and Tables

**Table 1:** Minimum inhibitory concentrations of isoniazid and rifampicin against *M. tuberculosis* by the direct resazurin microplate assay and absolute concentration methods

Absolute concentration method	Resazurin Microplate Assay Isoniazid (μg/mL)					

	< 0.0078125	0.015625	0.03125	0.0625	0.125	0.25
Resistant (*n* = 10)	0	0	1	0	1	8
Susceptible (*n* = 41)	33	2	5	0	1	0
	
	**Rifampicin (μg/mL)**					
	
	**< 0.015625**	**0.03125**	**0.0625**	**0.125**	**0.25**	**0.5**
	
Resistant (*n* = 8)	0		1	0	5	2
Susceptible (*n* = 43)	28	9	6	0	0	0

**Table 2:** Susceptibility results for 51 *M. tuberculosis* strains obtained by the direct resazurin microplate assay and absolute concentration methods

ACM	Isoniazid		Rifampicin		Total
	
	R	S	R	S	
R	9	1	7	1	18
S	1	40	0	43	84
Total	10	41	7	44	

REMA: Resazurin microplate assay

ACM: absolute concentration method

R: resistant

S: susceptible

**Table 3:** Performance characteristics of the resazurin microplate assay method for detecting resistance to isoniazid and rifampicin

Antibiotic	Sensitivity (%)	Specificity (%)	Accuracy (%)	PVR (%)	PVS (%)
INH	90.0	97.6	96.0	90.0	97.6
RIF	88.0	100.0	98.0	100.0	97.7

PVR: positive value for resistant

PVS: positive value for susceptibility

**Table 4:** Prevalence of multi-drug-resistant *M. tuberculosis* by the absolute concentration and resazurin microplate assay methods

DST method	Mono resistant TB	MDR-TB
ACM	6 (12%)	6 (12%)
REMA	5 (10%)	6 (12%)
